# The effect of intraoperative imaging on surgical navigation for laparoscopic liver resection surgery

**DOI:** 10.1038/s41598-019-54915-3

**Published:** 2019-12-10

**Authors:** Andrea Teatini, Egidijus Pelanis, Davit L. Aghayan, Rahul Prasanna Kumar, Rafael Palomar, Åsmund Avdem Fretland, Bjørn Edwin, Ole Jakob Elle

**Affiliations:** 1 The Intervention Centre, Oslo University Hospital, Oslo, Norway; 2 Department of Informatics, University of Oslo, Oslo, Norway; 3 Institute of Clinical Medicine, University of Oslo, Oslo, Norway; 4 Department of Surgery N1, Yerevan State Medical University, Yerevan, Armenia; 5 Department of Computer Science, NTNU, Gjøvik, Norway; 6 Department of Hepato-Pancreatic-Biliary surgery, Oslo University Hospital, Oslo, Norway

**Keywords:** Cancer, Three-dimensional imaging, Computed tomography, Preclinical research, Surgical oncology

## Abstract

Conventional surgical navigation systems rely on preoperative imaging to provide guidance. In laparoscopic liver surgery, insufflation of the abdomen (pneumoperitoneum) can cause deformations on the liver, introducing inaccuracies in the correspondence between the preoperative images and the intraoperative reality. This study evaluates the improvements provided by intraoperative imaging for laparoscopic liver surgical navigation, when displayed as augmented reality (AR). Significant differences were found in terms of accuracy of the AR, in favor of intraoperative imaging. In addition, results showed an effect of user-induced error: image-to-patient registration based on annotations performed by clinicians caused 33% more inaccuracy as compared to image-to-patient registration algorithms that do not depend on user annotations. Hence, to achieve accurate surgical navigation for laparoscopic liver surgery, intraoperative imaging is recommendable to compensate for deformation. Moreover, user annotation errors may lead to inaccuracies in registration processes.

## Introduction

Liver is the most common site for metastasizing colorectal cancer, which is the third most common form of cancer in both males and females with approximately two million new cases and nearly one million deaths worldwide in 2018^1,2^. Metastatic liver tumors are more common than primary tumors and are the most common indication for liver resection in western world. Resection remains the only potentially curative treatment method. Treatment is carried out either as open surgery or as Laparoscopic Liver Resection (LLR)^3,4^. LLR is a minimally invasive procedure performed through small abdominal incisions, through which laparoscopes and extended surgical tools are inserted after inflation of the patient’s abdomen with carbon dioxide gas. This process separates peritoneal layers and is commonly referred to as pneumoperitoneum.

In LLR, pneumoperitoneum creates space to visualize abdominal organs with the laparoscope camera. However, pneumoperitoneum also increases pressure in the abdomen, which causes the organs to deform^5–8^. Conventionally, LLR are performed using laparoscopic ultrasound (US) intraoperatively to guide the surgery. Preoperative images, such as computed tomography (CT) and magnetic resonance imaging (MRI), are also used for decision making as well as for surgical planning^9^. Through segmentation and reconstruction processes, these images can show relevant anatomical structures in 3D. Deformations such as pneumoperitoneum complicate the anatomical correlation with the CT and MRI scans that the surgeons perform during the surgery. This could lead to misinterpretation of the positions of major blood vessels or of the tumor within the tissue. Moreover, time between preoperative imaging and surgery has a negative effect on utility of the medical images and increases patient morbidity, due to unnecessary surgery^10^. For these reasons, surgeons rely on US for intraoperative guidance and detecting changes from preoperative imaging.

**Figure 1:**
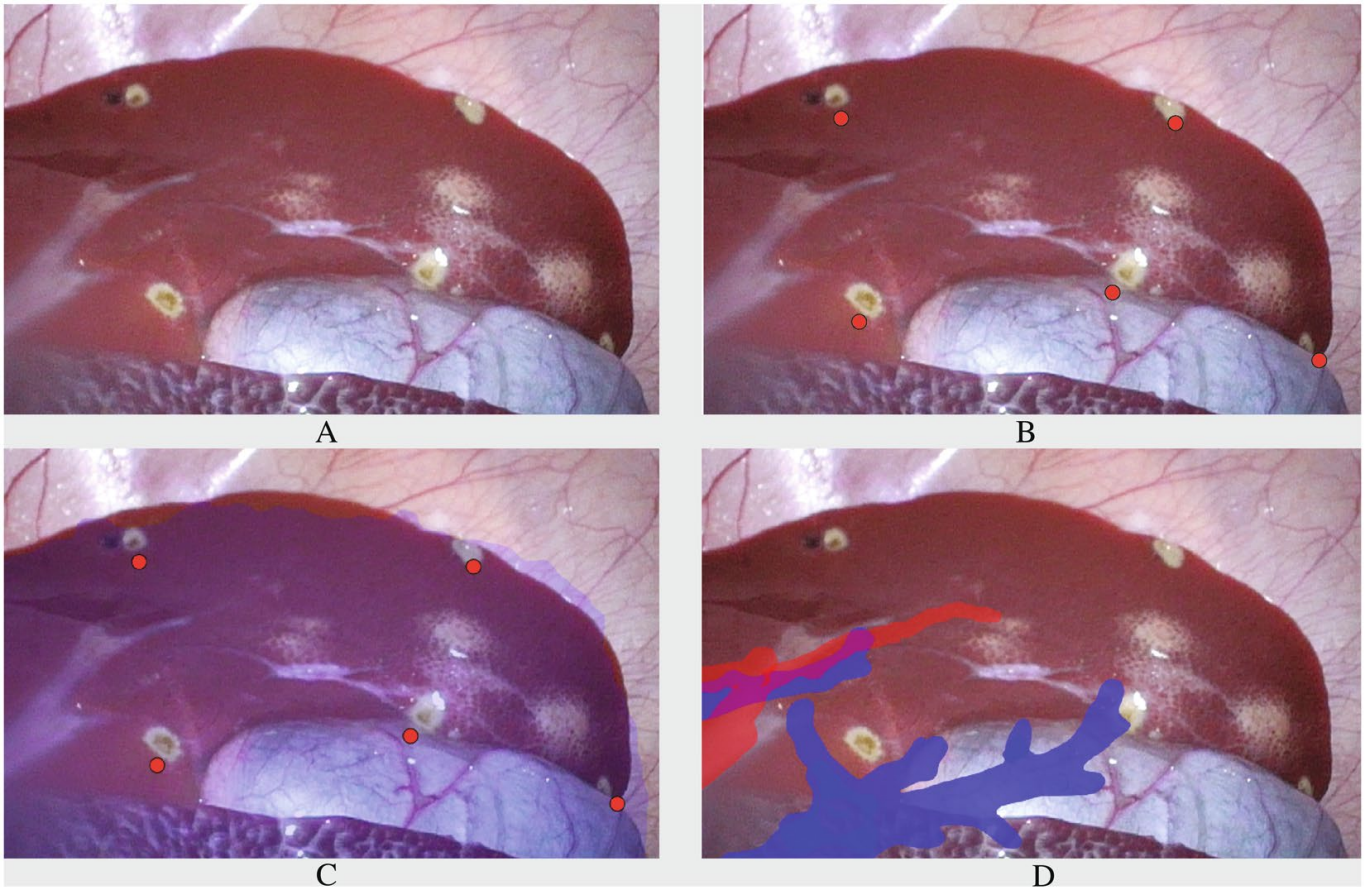
Laparoscopic liver frames from *in-vivo* study. (**A**) Is the original image, (**B**) shows the reprojected cauterization point centroids, used for TRE assessment, (**C**) shows the reprojection of the liver parenchyma and (**D**) shows some branches from hepatic and portal veins as AR. The AR frames in (**C**,**D**) show the error of the overlay.

As an aid to the surgeons, image-guided surgery (IGS) systems can be used to provide navigation. IGS utilizes computer-based systems to provide image fusions and tool guidance to the lesions. IGS display this navigation to surgeons either through 3D models on separate monitors or overlaid as augmented reality (AR), as shown in Fig. 1. Hybrid operation rooms (OR) are novel surgical suites which allow multiple imaging modalities to be used during surgical procedures, for example, using intraoperative sliding door CT scanners. These suites combine diagnostics, treatment and evaluation of surgical outcome in the same OR. With respect to liver surgery, CT and cone-beam computed tomography (CBCT) are used under interventional procedures for guidance of needles to a target in the liver e.g. biopsies, percutaneous biliary stenting, ablation treatments^11^. However, within LLR, intraoperative CT scanning is not in use yet in the regular clinical workflow because of the scarcity of Hybrid ORs. Through *in vivo* assessment of AR accuracy attained with state-of-the art algorithms for IGS, this study aims to test if intraoperative imaging is necessary for accurate surgical navigation for LLR. Moreover, since liver navigation systems make use of input from clinicians^12,13^, this study assesses how user annotations effect accuracy of IGS navigation. This study analyses the following hypotheses:

- Intraoperative imaging allows AR surgical navigation to be more accurate compared to preoperative imaging.
- Annotations performed by medical doctors can cause statistically significant differences in terms of accuracy in the surgical navigation.
- Fiducials remove user-induced error and significantly increase the accuracy of surgical navigation.
- A higher number of fiducials can increase the accuracy of surgical navigation, up to a certain point.

## Results

### AR is more accurate with intraoperative imaging

**Figure 2:**
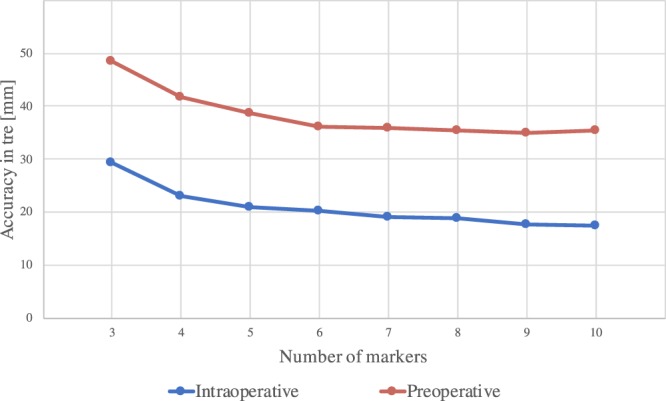
Average TRE in [mm] (±*σ*) of AR using intraoperative and preoperative 3D with user-defined markers with different number of markers (N) used for registration.

The Target Registration Error (TRE) for both pre- and intraoperative based AR are reported in Table 1. TRE is averaged across: four trials, number of user annotated markers for registration and ten repeated registration processes, evaluating a total of N = 3200 AR frames. A univariate ANOVA (repeated measures) was performed on TRE to verify if there is a difference between pre- and intraoperative TRE, across variability of the number of markers. The main effect for change of imaging modality yielded significant results, with an F ratio of F(1, 3198) = 198.798 and p = 1,001 * E-320 (p << 0.05), indicating a significant difference between accuracy in TRE using intraoperative imaging (*μ* = 19.04, *σ* = 10.26, n = 1600) and preoperative imaging (*μ* = 38.37, *σ* = 13.98, n = 1600), as shown in Fig. 2.

**Table 1:** Average TRE in [mm] (±*σ*) of AR using intraoperative and preoperative 3D with user-defined markers with different number of markers (N) used for registration.

N	Intraoperative	Preoperative	*P*-value
3	29.28 ± 8.08	48.60 ± 13.92	<0.001
4	23.11 ± 5.90	41.77 ± 9.35	<0.001
5	20.89 ± 6.23	38.79 ± 8.56	<0.001
6	20.33 ± 5.66	36.23 ± 7.08	<0.001
7	19.22 ± 5.15	35.87 ± 7.55	<0.001
8	18.79 ± 4.98	35.48 ± 6.78	<0.001
9	17.72 ± 4.71	34.97 ± 7.58	<0.001
10	17.51 ± 3.95	35.35 ± 7.98	<0.001
**Mean**	**19.04** ± **10.26**	**38.37** ± **13.98**	<**0.001**

### User induced error

#### Annotations between users can lead to significant changes in accuracy

**Figure 3:**
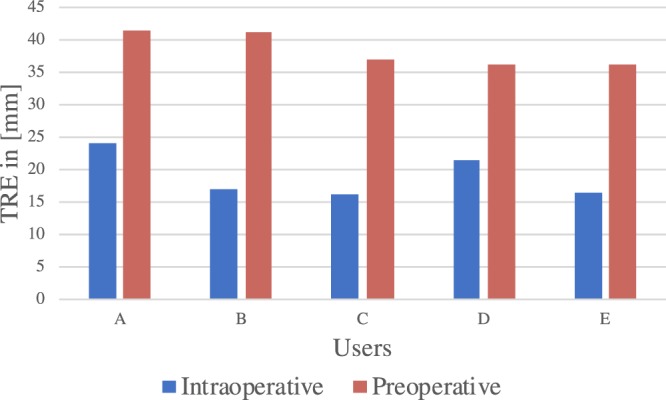
TRE between users for preoperative and intraoperative images, significant differences were found between users, demonstrating how user influence can cause significant differences.

Two univariate ANOVAs (repeated measures) were performed on TRE, one for intraoperative and another for preoperative data to verify if there is a difference between TRE between users. Based on Fig. 3, with respect to intraoperative data, Bonferroni post-hoc analyses between users A, D (24.18 ± 9.43 mm and 21.45 ± 12.68 mm, respectively) as compared to users B,C,E (17.05 ± 7.50 mm, 16.03 ± 11.83 mm and 16.50 ± 6.51 mm, respectively) deemed statistically significant results (p = 7.8908E-20, 2.7356E-25, 1.009E-22), moreover, also between A and D statistically significant differences were found (p = 0.000418), overall proving a significant difference between accuracy within users. With respect to preoperative data, Bonferroni post-hoc analyses between users A, B (41.56 ± 14.46 mm and 41.20 ± 14.53 mm, respectively) as compared to users C,D,E (36.85 ± 12.09 mm, 36.08 ± 12.83 mm and 36.08 ± 13.98 mm, respectively) also deemed statistically significant results (p = 0.000017, 9.4828E-7, 5.5311E-7) confirming a significant difference between accuracy within users also for preoperative imaging.

#### AR with fiducials (removing user induced error) improves AR

**Figure 4:**
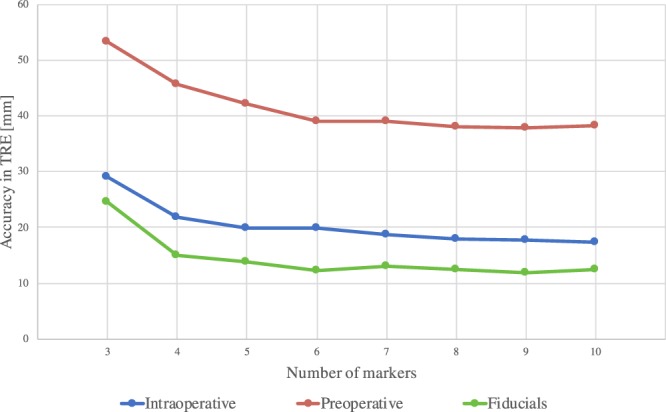
Average TRE in [mm] (±*σ*) of AR using intraoperative and preoperative images with a variable number (N) of either user-defined markers or inserted fiducials. This shown with different number of markers used for registration. N.B. Calculated over only three animals.

Inserted fiducials were used as user independent ground truth positions for the annotations performed by the clinicians. AR was tested using registered fiducials on three *in vivo* trials. One way repeated measures ANOVA, with an F ratio of F(2, 2639) = 1148.44 was used to compare TRE using user annotated registration markers and inserted fiducials. Results are represented in Fig. 4 and reported in Table 2. Bonferroni Post Hoc testing confirmed the previously established significant difference between accuracy in TRE of intraoperative imaging (*μ* = 20.30, *σ* = 10.48, n = 1200) and preoperative imaging (*μ* = 41.65, *σ* = 13.64, n = 1200) with p = 1,001*E-320 (p << 0.05); and also found significant differences with results using fiducials (*μ* = 14.38, *σ* = 10.89, n = 240) with intraoperative data p = 1.4339E-11 and preoperative data p = 1.2032E-189.

**Table 2:** Average TRE in [mm] (±*σ*) of AR using intraoperative and preoperative images with a variable number (N) of either user-defined markers or inserted fiducials.

N	Intraoperative	Preoperative	Fiducials	*P*-value (Intra vs Fiducials)
3	29.1 ± 7.95	53.22 ± 12.89	24.5 ± 26.85	1.000
4	21.85 ± 4.76	45.71 ± 6.85	14.93 ± 5.11	0.052
5	19.9 ± 5.98	42.06 ± 6.76	13.79 ± 4.67	0.047
6	19.78 ± 5.67	39.05 ± 5.39	12.22 ± 3.13	0.001
7	18.66 ± 5.26	39.03 ± 5.36	12.99 ± 3.7	0.011
8	18 ± 4.97	38.07 ± 5.19	12.36 ± 2.93	0.011
9	17.73 ± 5.2	37.88 ± 5.86	11.84 ± 2.54	0.011
10	17.37 ± 4.32	38.2 ± 6.49	12.43 ± 3.73	0.065
**Mean**	**20.30** ± **5.51**	**41.65** ± **6.85**	**14.38** ± **6.58**	<**0.001**

This shown with different number of markers used for registration. N.B. Calculated over only three animals.

**Figure 5:**
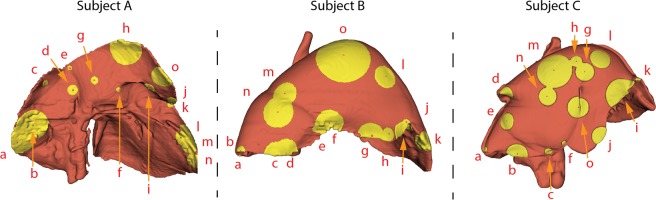
Fiducial Localization Errors, in [mm] computed across three animal trials based on the annotations from five clinicians. Annotations were performed on 3D liver models from intraoperative CT scans. Labeled red spheres indicate position of the fiducials and yellow areas cover median user-induced error for the specific marker.

Moreover, analysis of Fiducial Localization Error (FLE), as the difference between positions annotated by the medical doctors and the inserted fiducials, conducted across 375 distances, resulted in a user-induced annotation error of 16.81 ± 12.92 mm using intraoperative imaging (results are reported in Table 3). Moreover, across users inter-user variability was 16.89 ± 9.81 with respect to intraoperative imaging and 24.51 ± 12.81 mm for preoperative imaging (67% more variability in location annotation when using preoperative imaging). In terms of AR inaccuracy, FLE caused an increase of TRE of 6.69 ± 3.95 mm or 32.96% (visible in Table 2) with respect to intraoperative imaging. A representation of distribution of user-induced FLE across users is visible in Fig. 5 for the three trials in which FLE was evaluated.

**Table 3:** Fiducial Localization Errors, in [mm] computed across three animal trials based on the annotations from five clinicians.

Subject	a	b	c	d	e	f	g	h	i	j	k	l	m	n	o	Mean
A	63.3	56.5	11.4	18.2	8.6	13.0	18.3	54.6	21.3	16.7	16.3	38.6	34.9	31.6	37.6	29.4
B	6.3	9.6	41.8	42.9	25.1	12.2	34.2	46.2	21.8	30.7	6.1	38.9	30.3	38.4	89.5	31.6
C	12.5	36.3	13.6	24.1	28.1	8.7	29.9	19.8	58.0	31.4	9.0	35.3	57.5	28.7	34.4	28.5

Annotations were performed on 3D liver models from intraoperative CT scans. Labeled red spheres indicate position of the fiducials and yellow areas cover median user-induced error for the specific marker.

#### Increased number of registration markers reduced target registration error

**Figure 6:**
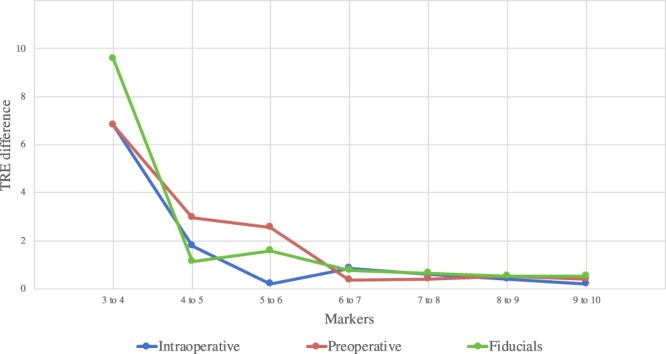
Decrease of TRE in [mm] when increasing number of markers (N) for image-to-patient registration, significance levels comparisons between pairs of number of markers are in between brackets (p-values).

A decrease of TRE follows an increase of the number of registration markers used (as can be seen in the diagrams in Figs. 2 and 4). Mean differences and significance between the TRE using different numbers of registration markers are reported in Table 4, for both user-defined and fiducial markers. Student’s t-tests performed for marker intervals resulted in non-significant differences between the use of 4 to 5 registration markers for both user-defined intraoperative and inserted fiducials, and non-significant differences were found after comparing 5 to 6 user-defined registration markers for preoperative models (as can be inferred from Fig. 6).

**Table 4:** Decrease of TRE in [mm] when increasing number of markers (N) for image-to-patient registration, significance levels comparisons between pairs of number of markers are in between brackets (p-values).

N	Intraoperative	Preoperative	*P*-value
3 to 4	6.8 (<0.001)	6.8 (<0.001)	9.56 (0.011)
4 to 5	1.77 (0.211)	2.97 (0.027)	1.14 (0.762)
5 to 6	0.19 (0.598)	2.55 (0.600)	1.56 (0.150)
6 to 7	0.86 (0.287)	0.37 (0.826)	0.763 (0.242)
7 to 8	0.59 (0.948)	0.38 (0.987)	0.624 (0.169)
8 to 9	0.38 (0.507)	0.51 (0.646)	0.524 (0.225)
9 to 10	0.19 (0.506)	0.38 (0.085)	0.529 (0.115)

## Discussion

**Figure 7:**
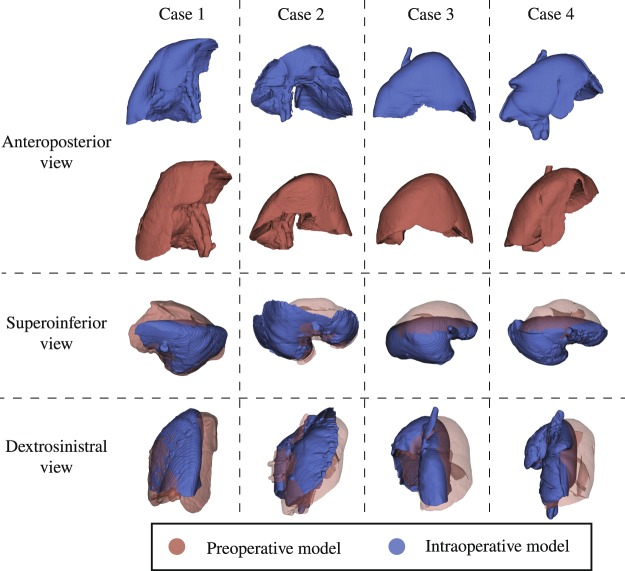
Difference between pre- and intraoperative shapes of the liver post pneumoperitoneum.

Based on the conducted study, there is a clear and significant difference between the accuracy attainable using the intraoperative CT scan against using the preoperative CT scan to perform AR. This is due to the liver deformation due to pneumoperitoneum, which can be very large and is clearly visible in Fig. 7. The deformation causes incorrectly shaped segmentation models to be re-projected, hence, increasing TRE from 19.04 mm (intraoperative) to 38.37 mm (preoperative).

However, this study also suggests that the correlation positions between preoperative scans to laparoscopic images is more complex, as compared to using intraoperative scans: the distances, between annotations by users, for intraoperative was found to be 16.89 mm, as compared to the 24.51 mm for the preoperative. This means that 67% more variability between clinicians was committed when annotating a location on the liver surface in preoperative as compared to intraoperative images. This result was expected, because the intraoperative scan reproduces the same shape of the liver visible through the laparoscope camera, whereas the preoperative does not. An increase in AR accuracy may help surgeon perform more precise parenchyma-sparing liver resection, which is already an accepted treatment method^14,15^, while maintaining safe resection margins, which could result in fewer unexpected bleedings and local recurrences. Hence, visualization and understanding of the liver anatomy for surgical planning and procedure guidance are improved by up-to-date images. However, since this study was conducted on porcine models, it would be relevant to perform similar evaluations for clinical cases, to verify if the assumptions and the results presented in this study are valid also for clinical applications.

The results shown in this paper indicates that it is recommendable to acquire new volumetric images and make use of intraoperative updates, during laparoscopic surgery, whenever surgical navigation is performed.

With respect to image-to-patient registration, we have shown that user-induced error may cause significant differences between the accuracy of AR (this is shown in Fig. 3). This means that the anatomical spatial correlation that the surgeon performs when finding correspondent positions between the laparoscope camera and the CT scan may increase inaccuracy. As confirmation of this, compared to ground truth, users induced, on average, 6.7 mm error to the TRE of the AR. This suggests that registration methods which do not rely on the user and make use of intraoperative imaging, can result in a much lower TRE, therefore also in a more accurate surgical guidance. AR using fiducials resulted in an average TRE of 14.38 mm, whereas, for precise guidance, surgeons request a TRE closer to 6 mm. With measured accuracy, AR could be used for easier orientation in the operating field by visualizing underlying structures and should be complimented with ultrasound for precise navigation. An improved accuracy could be achieved for example by using surface reconstruction^16^, tracked laparoscopic ultrasound, injected fiducials for registration or a combination of the previous. Moreover, based on Fig. 5, it appears that locations in the middle and on the top of the liver are more inaccurately annotated due to large distances to relatable structures or edges. These inaccuracies may lead to a less precise AR, so it is preferable to have registration markers at the edges of the liver, in case fiducials are not used.

When using fiducials for registration, the accuracy for the AR was shown to be dependent on the amount of registration markers used to compute image-to-patient registration, however, up to a plateau. Within this study, the plateau for TRE was reached at different numbers of markers depending on the images and annotations used. Data shows that for preoperative models using 5 or more markers achieves lowest TRE and 4 or more for the intraoperative, both with user annotations and inserted fiducials (see Figs. 4 and 6). This means that using five number of markers should be suggested for accurate and time-effective registration, which would allow optimal laparoscopic AR liver surgeries. Moreover, we found that it is not recommendable to add more registration markers, but it may instead be preferable to re-acquire the landmarks. It is important to mention that porcine and human liver present anatomical differences, such as the fact that the porcine model presents a thinner and more mobile liver, which might be more sensitive to deformations due to pneumoperitoneum, position and breathing. Thus, this study should be repeated through clinical evaluations. Due to mobility of the liver, the liver underwent deformation during insertion of the fiducials. This was compensated for with model-to-model registration, however, this transformation might have introduced some inaccuracy in the ground truth positions. This could be improved by using fiducials which can attach directly to the liver surface, without the need of puncturing the liver parenchyma. With respect to annotation of the 3D models, these were performed on a 2D monitor using a mouse. Even with the ability to manipulate the model, user’s spatial understanding of the model might have been limited and could have been improved using a 3D monitor or a mixed reality device. Visualization of 3D models in mixed reality improves spatial understanding of patient-specific organ anatomy while reducing time, as compared to conventional imaging (as shown in^17^). Through more accurate and appropriate visualization of the volumes and precise placement of registration markers, the difference between where the user wanted to position the markers and where they were placed could have been minimized. Other limitations to this study include the inaccuracy in annotation of ground truth positions to evaluate TRE in the laparoscopic frames: centers of the cauterization markers were manually annotated. Another was the precision of sampling registration markers using laparoscopic optically tracked surgical instruments on the liver surface: breathing motion on the porcine liver and user stability while sampling the position. These inaccuracies were partially compensated by longer acquisition and averaging the tracked positions across the sample. These errors might have introduced more inaccuracies in the evaluation of the TRE. The method presented in this study is also limited by subject positioning: the OR table needs to be positioned at zero degrees with respect to the CT gantry. Hence, if other table orientations were to be preferred, additional deformations to the liver would be present and would need to be compensated for. Examples of solutions for non-rigid deformation are available in the literature^18,19^. However, these methods rely on estimated viscoelastic parameters, which change inter-patient with different stages of fibrosis, as well as estimation of the boundary conditions^20^. For these reasons, to obtain accurate non-rigid registration, elastography may need to be conducted for each patient. In conclusion, this study shows significant improvements in AR accuracy when using intraoperative imaging and that user annotations for registration caused 33% more inaccuracy. Hence, this study indicates, that to achieve precise surgical navigation for laparoscopic liver surgery, intraoperative imaging could compensate for some of the deformations. Furthermore, user annotations may lead to increases of inaccuracy in augmented reality. Clinical trials would need to be conducted to validate the findings of this study in patients.

## Materials and Methods

This study assesses the accuracy of reprojection of volumetric data to the laparoscopic camera. Section 2.1 describes the experimental protocol and equipment used. Section 2.2 details the implementation of the AR for LLR. Section 2.3 describes the methods used to evaluate AR accuracy and Section 2.4 describes the dataset used for this study.

### Experimental protocol

#### Operation room and equipment

A Hybrid OR, equipped with a SIEMENS SOMATOM CT scanner as well as an ARTIS pheno CBCT, was used to perform the LLR and acquire medical images directly on the operating table. A Flex3D Olympus^®^ stereo laparoscope camera was used for video acquisition. To cancel out the flexibility function of the laparoscope camera, a 3D printed shaft was used throughout the experiment. De-interlaced and synchronized raw video was acquired from the laparoscope using a Blackmagic^®^ DecLink Duo. Surgical instrument tracking of the laparoscope camera and laparoscopic instruments was performed through optical tracking with an NDI^®^ Polaris Spectra. The OR was prepared with conventional equipment for laparoscopic liver resection with the addition of equipment needed for IGS.

#### CT image acquisition

Image acquisition of the CT scans were performed on operating table in the OR. CT images were acquired with injection of contrast, Ombipaque 350 mg/ml. Subjects were positioned supine with a slight flexion and rotation with respect to the OR table. Imaging through the sliding CT scanner was performed while interrupting breathing to reduce imaging artifacts due to breathing motion. To simulate current clinical scenarios, images were acquired both preoperatively (without pneumoperitoneum) and intraoperatively (after pneumoperitoneum). Pneumoperitoneum was stabilized at 13 mmHg for all intraoperatively acquired images.

#### Segmentation

CT scans taken before and after pneumoperitoneum were used to create liver parenchyma segmentations semi-automatically using ITK-SNAP followed by manual corrections^21,22^. These segmentations were later exported into 3DSlicer^23^ and reconstructed into a 3D model.

### Augmented reality

IGS combines medical image segmentations and surgical instruments tracking onto the visual of the laparoscope camera. When both image sources are overlaid, this technology is known as AR. Within this study, AR was obtained through a combination of computer vision algorithms: hand-eye calibration and image-to-patient registration.

#### Hand-eye calibration

Hand-eye calibration is conventionally used in computer vision to find the transformation matrix between a “hand” (a robotic arm) and an “eye” (camera source). Within the IGS field, hand-eye calibration translates into finding the transformation between instrument tracking sources (the optical tracking system in our study) and the pose of the laparoscope camera.

**Figure 8:**
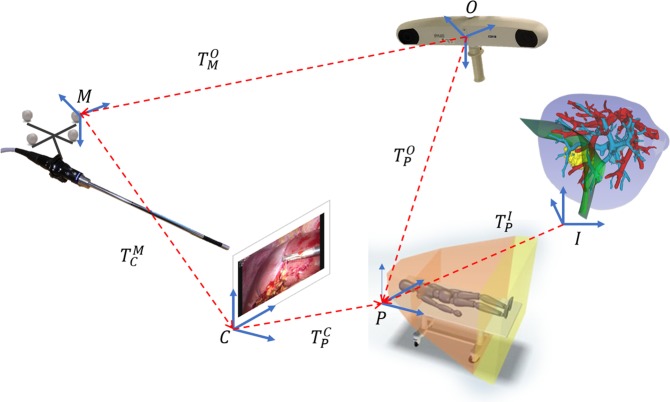
Diagram of transformations used to perform AR for liver surgery.

Within this study, hand-eye calibration was computed according to^24^. Based on the transformation diagram for this study (Fig. 8), through multiple posed images of a laser printed calibration plate (96 dark laser printed circles with 2.5 mm in diameter over a white background) with four optical markers at machined locations (manufactured by Cascination AG^®^), hand-eye calibration (transform ${T}_{C}^{M}$) was computed as follows:

1 $$[\begin{array}{c}{[\begin{array}{cc}I\otimes {({R}_{O}^{M})}^{-1} & {Z}_{\mathrm{9,3}}\\ {Z}_{\mathrm{3,9}} & {({R}_{O}^{M})}^{-1}\end{array}]}_{1}\\ \vdots \\ {[\begin{array}{cc}I\otimes {({R}_{O}^{M})}^{-1} & {Z}_{\mathrm{9,3}}\\ {Z}_{\mathrm{3,9}} & {({R}_{O}^{M})}^{-1}\end{array}]}_{N}\end{array}]\times [\begin{array}{c}vec({R}_{C}^{M})\\ {t}_{C}^{M}\end{array}]=[\begin{array}{c}{[\begin{array}{c}vec({R}_{C}^{O})\\ {t}_{C}^{O}+{({R}_{O}^{M})}^{-1}\cdot {t}_{O}^{M}\end{array}]}_{1}\\ \vdots \\ {[\begin{array}{c}vec({R}_{C}^{O})\\ {t}_{C}^{O}+{({R}_{O}^{M})}^{-1}\cdot {t}_{O}^{M}\end{array}]}_{N}\end{array}]$$

where is the Kronecker Product operator and *Z*_3,9_ and *Z*_9,3_ are matrices of zeros with 3 rows and 9 columns and 9 rows and 3 columns respectively, vec(R) represents the 3 × 3 matrix vectorized in column notation, and R and t are respectively Rotation matrices and translation vectors. In this paper superscript indicates the coordinate system with respects to which the transformation is applied, and subscripted indicates the coordinate system towards which the transformation is applied. Moreover, all transformations are homogeneous 4 × 4 matrices.

#### Image-to-patient registration

Image-to-patient registration computes the transformation between the image space (the CT scan) and the patient position and orientation while on the OR table. This transformation in Fig. 8 is represented by transformation matrix ${T}_{P}^{O}$. Most approaches in the literature^13,25^ compute rigid transformations. The same was performed throughout this study. Optical tracking was used to track the tip position of a rigid laparoscopic surgical instrument (Stryker^®^ needle holder), which was used to sample positions directly on the liver surface during surgery^26^. Image-to-patient registration was computed through singular value decomposition between the sampled points and the correspondent positions annotated on the medical images.

After computing hand-eye calibration and image-to-patient registration, for each frame acquired by the laparoscope camera, Eq. (2), which follows the transformation diagram in Fig. 8, was used to perform the overlay of the annotated and segmented structures:

2 $${T}_{I}^{C}={M}_{I}\cdot {({T}_{C}^{M})}^{-1}\cdot {({T}_{M}^{O})}^{-1}\cdot {T}_{P}^{O}\cdot {({T}_{P}^{I})}^{-1}$$

where transformations ${T}_{P}^{O}$ and ${T}_{M}^{O}$ are provided by optical tracking and represent respectively the transformation to the patient coordinates sampled with the surgical instrument and the position and orientation of the markers on the laparoscope camera; ${T}_{I}^{P}$ is provided by image-to-patient registration and *M*_*I*_ is the intrinsics of the camera computed through camera calibration^27^.

### Evaluation of accuracy

In the following chapter, the authors describe procedure with which the AR system was tested *in vivo*. Measures of accuracy evaluated in this study were Target Registration Error (TRE) and Fiducial Localization Error (FLE)^28,29^ obtained in through four pre-clinical porcine model trials. For each trial, 100 augmented reality images were randomly selected from the laparoscopic video.

Cauterization markers were performed on the liver surface and were used for image-to-patient registration and evaluation of the TRE (all 15 marks were visible within each of the 100 frames), as performed in^30^. To compute FLE, fiducials were inserted, in three trials, at the location of each cauterization mark. All cauterization marks within each frame were manually annotated to compute the TRE (totaling 400 annotated frames with two to three cauterization marks per frame on average). The error in AR is represented by the distance between the center of the reprojected AR blue dots to the manually annotated centroids of the cauterization marks, as shown in Fig. 1. Image-to-patient registration was performed using annotations performed by five medical doctors, whom annotated the 15 cauterization marks on both pre- and intraoperative images. To evaluate the effect of using a variable number of marks to perform image-to-patient registration, three to ten markers were used for each trial, with random sampling of the markers, repeated ten times. Hence, a total of 64,000 AR frames were computed for each medical doctor’s annotations, totaling 320,000 AR frames for TRE evaluation. FLE was instead computed as the Euclidean distance between the inserted fiducial and the correspondent cauterization mark that the medical doctors annotated. This resulted in a total of 375 distances evaluated for FLE.

#### Target registration error

Target Registration Error is a measure of accuracy which represents the registration error in locations at a distance from the registration targets^28,29^. For IGS in AR systems, this equates to computing the error in visualizing structures that are not used to perform image-to-patient registration. To evaluate the error on the *in vivo* studies, a total of 15 cauterization marks across the liver surface were created through an optically tracked laparoscopic monopolar (Aesculap^®^) for each trial. TRE can be visualized as the distance between the reprojected dots, visible in Fig. 1 in frame *(B)*, and the positions of the cauterization marks. The TRE is also perceivable when reprojecting as AR the liver parenchyma (Fig. 1 in *(C)*) or blood vessels (Fig. 1 in *(D)*. Each of the 15 cauterization marks were annotated by the clinicians on the 3D models from pre- and intraoperative CT scans using 3D Slicer. To remove user inaccuracy due to human interpretation during annotations, fiducials were inserted at the location of the cauterization marks. After the insertion of the fiducials, an additional intraoperative CT or CBCT was performed, in which the fiducials were clearly visible. Liver parenchyma and fiducials are segmented from the images and processed into 3D models. To compensate for changes made during insertion of the fiducials and for new origins of the intraoperative CT scans, surface-to-surface registration is performed on models with and without fiducials, using Meshlab^31^ and 3D Slicer^23^. Position of fiducials are annotated on the intraoperative model. Cauterization marks were used in random order and selection to perform image-to-patient registration and to evaluate TRE. Since some cauterization markers were used to compute the registration, the markers which were used in the registration process were excluded from TRE computation. A variable number of markers was used to perform image-to-patient registration, ranging from 3 to 10 markers, to see whether this would lead to a better accuracy for the AR. TRE was computed as the distance between the center of the cauterization mark manually annotated for each frame and its correspondent reprojected position as AR. To provide the user with an error in millimeters, and not in pixels (since it would not provide an evaluation of the accuracy in the depth), the inverse of the intrinsics calibration matrix was used, as was performed in^32^.

#### Fiducial localization error

Fiducial Localization Error is the error committed in picking the position of a fiducial point^28,29^. To perform image-to-patient registration, the cauterization marks were sampled with the laparoscopic tool (patient coordinates), however, their correspondent equivalents in the image space were manually annotated by the surgeon while looking at the laparoscopic video. Hence, with respect to image-to-patient registration, FLE is the error committed by the surgeon when selecting a fiducial on the 3D models from CT scans. The annotated inserted fiducials provide ground truth positions for FLE. FLE was computed as the Euclidean distance between the positions annotated by the clinicians on the CT scan and the ground truth positions from the fiducials. This error is displayed for a single animal trial in Fig. 5, where the yellow area represents the FLE committed on average for each marker (represented by the red dots).

### Dataset and statistics

#### Dataset

Four trials on porcine models were included in this study. The animal study was approved by the National Animal Experimentation Board [project ID: 12633], and carried out in accordance with Norwegian regulations concerning use of animals in experiments [FOR-2015-06-18-761]. In this dataset, subjects weight ranged from 59.5 kg to 70 kg with liver volume ranging from 1593 *cm*^3^ to 2761 *cm*^3^. Aside from the tracking information, each data set includes: laparoscopic video recording with cauterized marks on the liver surface; CT images before and after pneumoperitoneum as well as imaging with inserted fiducials in three cases. Data was annotated by five medical doctors, with varying experience with liver surgery. Since the fiducials were inserted laparoscopically, FLE was computed only for annotations performed on the intraoperative scans, although the medical doctors performed annotations of the cauterization marks also on preoperative imaging (without pneumoperitoneum).

#### Statistical analyses

Statistical analyses were performed using SPSS (IBM^®^ SPSS Statistics for Windows, version 25.0, Armonk, NY, USA: IBM corp). All measurements are shown as mean with standard deviation based on the law of large numbers, even though small parts of the data is not normally distributed. Significance between TRE using different registration marks were calculated using One way and repeated measures ANOVAs with Bonferroni post-hoc testing. Significance between the TRE between different number of registration marks was calculated using Student’s t-test.
